# Three Siblings with Idiopathic Hypogonadotropic Hypogonadism in a Nonconsanguineous Family: A Novel *KISS1R/GPR54* Loss-of-Function Mutation

**DOI:** 10.4274/jcrpe.galenos.2019.2018.0230

**Published:** 2019-11-22

**Authors:** Özlem Nalbantoğlu, Gülçin Arslan, Özge Köprülü, Filiz Hazan, Semra Gürsoy, Behzat Özkan

**Affiliations:** 1 Dr. Behçet Uz Pediatric Diseases and Surgery Training and Research Hospital, Clinic of Pediatric Endocrinology, İzmir, Turkey; 2 Dr. Behçet Uz Pediatric Diseases and Surgery Training and Research Hospital, Clinic of Pediatric Genetics, İzmir, Turkey

**Keywords:** Kisspeptin, KISS1R, hypogonadotrophic hypogonadism, delayed puberty

## Abstract

Idiopathic hypogonadotropic hypogonadism (IHH) is a rare disease caused by defects in the secretion of gonadotropin releasing hormone (GnRH) or the action of GnRH on the pituitary gonadotrophes. *KISS1R* is one of the genes which, when mutated, cause IHH and mutations of this gene are responsible for about 2-5% of patients with normosmic IHH (NIHH). In this report, we present three siblings with NIHH due to a compound heterozygous *KISS1R* mutation. Genetic studies were carried out in the 14 year old index case with IHH and three siblings, two of whom were prepubertal. Genomic DNA was extracted from peripheral leukocytes and *KISS1R* gene was sequenced by using standard polymerase chain reaction amplification procedures. In molecular analysis of the index case, a compound heterozygous mutation was determined in *KISS1R* gene c.969C>A (p.Y323X) (known pathogenic) and c.170T>C (p.L57P) (novel). Mutation c.170T>C (p.L57P) was inherited from the mother while c.969C>A (p.Y323X) was inherited from the father. The same genotype was also found in two of the three siblings. A compound heterozygous mutation of the KISS1 gene, including one novel mutation, was found to cause NIHH and also incomplete puberty in a non-consanguineous family.

**What is already known on this topic?**

KISS1 and its receptor, KISS1R, (formerly called GPR54) play key roles in the initiation of puberty. Kisspeptin, a peptide encoded by the *KISS1* gene and its receptor are essential to stimulate gonadotropin releasing hormone secretion from the hypothalamus, which in turn stimulates pituitary gonadotrophin secretion to initiate puberty. Thus, the function of KISS-1 and KISS1R in the hypothalamus is critical for the onset and progression of puberty. Loss of function mutations in *KISS1R* gene can cause normosmic idiopathic hypogonadotropic hypogonadism (NIHH). To date, more than 20 different mutations have been reported. Most of these were loss of function mutations.

**What this study adds?**

A compound heterozygous mutation of the *KISS1R* gene was found to cause NIHH as well as incomplete puberty. In previous studies, the loss-of-functional mutations of *KISS1R/GPR54* which were inherited in an autosomal recessive manner were reported in consanguineous families. The cases reported herein were from a non-consanguineous family, illustrating a different phenotypic spectrum of KISS1R/ GPR54. We recommend genetic counselling for families with *KISS1R* mutations, even when there is no consanguinity.

## Introduction

Idiopathic hypogonadotropic hypogonadism (IHH) is a rare genetic disorder, which is caused by defects in the secretion of gonadotropin releasing hormone (GnRH) or the action of GnRH on the pituitary gonadotrophes (1). Increase in frequency and amplitude of the pulsatile secretion of GnRH is essential for the initiation of normal pubertal development. The failure of pulsatile secretion of GnRH from the hypothalamus leads to impairment of pubertal development and reproductive function, the clinical entity IHH. The clinical presentation of IHH may manifest as absent or incomplete puberty, cryptorchidism, small penis and infertility in males and amenorrhea, absence of breast development and infertility in females. IHH is divided into two major groups: Kallmann syndrome (KS) which is characterized with delayed puberty and impaired sense of smell and normosmic IHH (NIHH) (2). KS can show a wide variety of additional signs and symptoms. These include a failure of one kidney to develop (unilateral renal agenesis), abnormalities of bones in the fingers or toes, a cleft lip with or without an opening in the roof of the mouth (a cleft palate), abnormal eye movements, hearing loss, and abnormalities of tooth development (3). The incidence of IHH is approximately 10 in 100,000 live births and 60% of these patients have KS (3). NIHH results from the dysfunction of the normally situated GnRH neurons in the hypothalamus. Patients with NIHH typically do not have any accompanying congenital anomaly. To date about 50 genes have been reported to be associated with IHH (2). However, a smaller number of these genes are reported to be responsible in the pathogenesis of NIHH (1,2,4,5). Pathogenic mutations can be detected in about half of IHH cases (1,2). *KISS1R*, which encodes the kisspeptin receptor KISS1R, is one of the genes which causes NIHH, and mutations of this gene are responsible for 2-5% of patients with NIHH (5,6). To date, more than 20 different mutations have been reported. Most of these were loss of function mutations (7).

Kisspeptin is a peptide encoded by the *KISS1 *gene and its receptor, KISS1R, are essential to stimulate GnRH release from the hypothalamus which, in turn, stimulates pituitary gonadotrophins secretion to initiate puberty. Thus the genes *KISS-1 *and its receptor, *KISS1R*, (formerly called GPR54) play key roles in the initiation of puberty and the respective proteins KISS-1 and KISS1R and their function in the hypothalamus are critical for the onset and progression of puberty.

Here, we present three siblings from a non-consanguineous family with NIHH due to a compound heterozygous mutation including the previously reported c.969C>A (p.Y323X) and a novel c.170T>C (p.L57P) mutation in *KISS1R*.

## Case Report

The proband, a 14 year-old boy, was referred to our outpatient clinic due to lack of pubertal development. He was the first child of healthy, non-consanguineous, Turkish parents. He had three sisters whose ages were fourteen, twelve and five years. He was reported to have microphallus and bilateral undescended testicles in the newborn period. Bilateral orchiopexia was performed when he was one and a half years old. On physical examination, height was 165.3 cm [0.14 standard deviation score (SDS)], weight 62 kg (0.94 SDS) and bone age 14.0 years. He had typical signs of complete hypogonadism, including microphallus, enuchoid habitus (upper segment/lower segment ratio <0.9 and arm span >height) and lack of pubic and axillary hair. Both testicles were intrascrotal and the testis sizes were 3 mL, bilaterally. He had a normal sense of smell on olfactometry. No craniofacial stigmata or other morphological abnormalities were detected in the physical examination. His karyotype was 46,XY. Basal serum luteinizing hormone (LH), follicule-stimulating hormone (FSH), plasma testosterone (T), adrenocorticotropin, dehydroepiandrosterone sulfate and cortisol concentrations were determined by electro-chemiluminescence immunoassay (Table 1). An intravenous GnRH-stimulation test was also performed to obtain stimulated FSH and LH levels at 0, 20, 40 and 60 minutes, to confirm a diagnosis of hypogonadotropic hypogonadism. Magnetic resonance imaging of the central nervous system revealed normal findings.

The oldest sister, who was also 14 years old at the time of diagnosis, had breast development corresponding to Tanner stage 2. She had no pubic and axillary hair and was premenarcheal. Her bone age was appropriate at 14 years. Her breast development first appeared at age 10 years and after that no further progression in pubertal stages had occurred. Pelvic sonography revealed a uterus (47x18x11 mm) and two small ovaries (24x18x14 and 20x18x14 mm). Hormone assays were: basal FSH: 4.06 mIU/mL, LH: 1.21 mIU/mL and estradiol: 14 pg/mL.

The second sister of the proband was 12 years old and had no sign of pubertal development. Pelvic sonography showed a small uterus and small ovaries (uterine size was 25x6.5x13 mm; right ovary was 9.5x7x13 mm, left ovary was 6.5x10.5x15 mm). Evaluation of basal and GnRH stimulated hormone levels confirmed incomplete puberty. The youngest sister, who was 5 years old, had Tanner stage 1 breast development and a prepubertal hormone profile.

Karyotype analysis of all three sisters were 46,XX.

**Figure 1:**
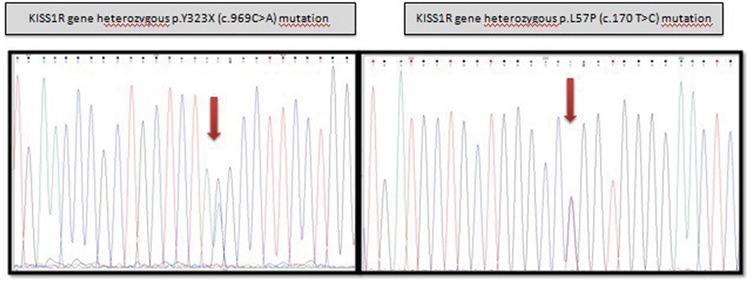
*KISS1* gene mutations detected in the index patient

Genomic DNA was extracted from peripheral leukocytes and the promoter region, the three coding exons and exon-intron boundries of the *KISS1R *gene (NM_032551) were amplified by polymerase chain reaction and sequenced. In the index case a compound heterozygous mutation in the *KISS1R* was present, comprising a nonsense variant (c.969C>A, p.Y323X) which was known as an inactivating mutation to cause NIHH and a novel missense variant (c.170T>C, p.L57P; see Figure 1). This novel missense variant was evaluated for functional impact using a variety of *in-silico *prediction tools including SIFT, PolyPhen-2 and Mutation Taster which supported a disease-causing effect of this mutation (8,9,10). Molecular analysis of the parents showed that both parents were heterozygous carriers. While the mutation c.969C>A (p.Y323X) was inherited from the father, the novel c.170T>C (p.L57P) variant was inherited from the mother. Genetic analysis of the older two sisters, who were 12 and 14 years old, revealed the same compound heterozygous mutation, whereas the genetic analysis of the youngest sister revealed a normal karyotype and normal KISS1R sequence. Clinical and hormonal characteristics of all cases, including the proband, are shown in Table 1.

Informed consent from the parents of the patients was obtained for publication.

## Discussion

Timing of onset of puberty is related to increased GnRH pulses which in turn, activate the increase in gonadotropin and sex hormone levels. Interaction of kisspeptins and their corresponding receptors has been reported to have a critical role in initiation and development of puberty (2). Inactivating mutations of *KISS1R *lead to NIHH (11,12,13).

Kisspeptin, which is a very potent stimulator of GnRH secretion, is secreted from neurons located in two different parts of the mammalian hypothalamus, the preoptic area and the arcuate nucleus (14). It is not only a potential stimulator of GnRH but also a mediator of positive and negative feedback effects on sex steroids (15).

More than 20 mutations in the *KISS1R *(*GPR54*) gene have been previously described and these mutations have variable clinical manifestations (16,17).

Recently, Topaloglu et al (18) reported an inactivating mutation of *KISS1 *causing complete NIHH in a large consanguineous family from Turkey. The probound was 14.9 years-old. She had no breast development and her pelvic ultrasonography revealed a hypoplastic uterus and ovaries lacking follicles. The affected three sisters of the proband had no spontaneous breast development. All four affected sisters were otherwise healthy and had a normal sense of smell.

Demirbilek et al (19) identified a homozygous nonsense mutation, c.C969A (p.Y323X) in the *KISS1R *gene in three non-consanguineous families with NIHH. One male presented with absence of pubertal onset and severe penoscrotal hypospadias and cryptorchidism. Two other males had absence of pubertal onset. Two of four female cases required replacement therapy for pubertal onset, while the other two females had spontaneous pubertal onset but incomplete maturation. A similar nonsense mutation, at position 969 of the nucleotide sequence in the *KISS1R *gene (c.C969>A) located on the short arm of chromosome 19 (19p13.3), has been reported in a case of normosmic IHH in a female patient from a consanguineous family (1). This nonsense mutation results in the creation of a premature stop codon that leads to incomplete production of the kisspeptin receptor. This truncated KISS1R protein fails to signal the release of GnRH from the hypothalamus.

Nimri et al (16) reported two highly consanguineous families of Israeli-Arab origin. Among these, some had evidence for complete hypogonadotropic hypogonadism. Cryptorchidism and a relatively short penile length were noted in all male patients at birth. A novel loss-of-function mutation in the *GPR54 *gene in six members of the family was identified (16).

Breuer et al (17) described a novel, severe homozygous *KISS1R *splice site mutation in three siblings in a consanguineous Palestinian family with IHH. They had normal neonatal external genitalia, but presented with no pubertal development, normosmia and a low response to GNRH stimulation.

*KISS1R *mutations which have been reported previously include point mutation, deletion, insertion, acceptor splice site mutation and missense mutation. Hereby, we described a compound heterozygous mutation in *KISS1R *gene in a non-consanguineous family. One of these was a known pathogenic nonsense variant (c.969C>A, p.Y323X) and the other was a novel missense variant (c.170T>C, p.L57P). The proband had NIHH, whereas his two sisters had incomplete pubertal development and the other sister was prepubertal. Previously described inactivating mutations associated with the *KISS1R *gene have been homozygous from consanguineous marriages. In this report, for the first time, we described *KISS1R *gene mutation in a non-consanguineous family. Thus, we have shown that kisspeptin receptor insufficiency can manifest as different clinical entities. In this study, we report that although three siblings have the same inactivating compound heterozygous mutation, one of them has incomplete puberty and amenorrhea, while the remaining two have NIHH. Different phenotypes can be obtained with the same mutation. In conclusion, we report a compound heterozygous mutation of the *KISS1R *gene causing normosmic IHH and incomplete puberty in siblings. In previous studies, the loss-of-functional mutations of *KISS1R/GPR54*, which were inherited as autosomal recessive mutations, are reported in consanguineous families. We identified these mutations in a non-consanguineous family, a finding which illustrates the different phenotypic spectrum of *KISS1R/GPR54*. We recommend genetic counselling for families with *KISS1R *mutations, even in non-consanguineous families.

## Figures and Tables

**Table 1:**
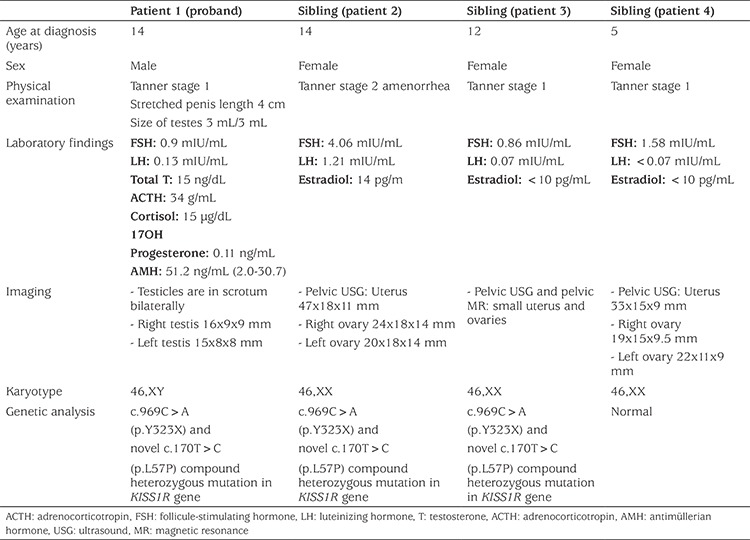
Clinical and hormonal characteristics of the proband and siblings
